# The Origin and Progress of the Malignant Cholera at Manchester

**Published:** 1834-01-01

**Authors:** 


*The Origin and Progress of the Malignant Cholera in Manchester, considered chiefly in their Bearing on the Contagiousness and the Secondary Causes of the Disease. To which are added, some Remarks on the Treatment. With an Illustrative Chart.* By HENRY GAULTER, M.D.—London, 1833. 8vo. pp. 206.

PREVIOUSLY to the publication of Dr. Gaulter's work, no account of the first visitation of the cholera at "the capital of the manufacturing districts" had made its appearance. That a place of such importance should remain unnoticed in the annals of cholera, while each petty village in the kingdom which has suffered from the disease can furnish its chronicles on the subject, was more than our author could endure; and he writes, therefore, for the future historian, of the most extraordinary epidemic that has happened for many generations.

At the commencement of a work which has for its subject the consideration of topics which have been the cause of much controversy, it would not be unreasonable to expect some observations respecting the many causes which tend to lead us into error. Dr. Gaulter says that

"The poor are habitually inexact: they omit, from stupidity, the most essential point of an inquiry, unless led to it by a direct question; or they answer as they suppose you wish them to answer; or else they wilfully deceive; nor is it difficult to imagine many powerful motives for concealment or falsehood in such an investigation. It is often, in short, only by a separate examination and cross-examination of the patient, relations, and neighbours, managed with all the astuteness of a lawyer, and that too after the first alarm has passed away, and the tranquillity of the house or neighbourhood has been restored, that the true particulars of the origin of any individual case can be correctly learned. Nor are the poor the only deceivers. The inquirer is himself, from an unconscious bias, not seldom in pursuit less of the truth, whatever it may be, and wherever it may lead, than of some preconceived opinion or exclusive system. He is a contagionist, and puts but one kind of question, or hears but one class of answers: he is an adversary to contagion, and interrogates only on localities and miasm." (P. 3.)

Now, although there is nothing in the above statement to which our readers would refuse their assent, we are strongly inclined to believe that they would more readily admit these truths in their study than apply them in their practice. Most of the vague theories and strange doctrines which abound in the history of medicine owe their origin to the overlooking such plain and simple truths; and, although the winnowing-fan of time has to a great extent separated the good from the bad, we cannot but look with astonishment at the mass of rubbish which in its day has been dignified with the title of a medical theory. There is much more required of a medical man than the qualifications for which he offers his certificates on entering his profession, if he would use that profession as a means to benefit others, and give pleasure to himself: his study must be directed to the mind as well as to the body, or he will soon have cause to repent his ignorance.

Theory-building has been beautifully described in the "Novum Organum," under the section "Of the False Images, or Idols, of the Mind."—"When the mind is once pleased with certain things, it draws all others to consent, and goes along with them; and, though the power and number of instances that make for the contrary are greater, yet it either attends not to them or despises them, or else removes and rejects them by a distinction, with a strong and pernicious prejudice to maintain the authority of its first choice unviolated. And, though the mind were free from this delight and vanity, yet it has the peculiar and constant error of being more moved and excited by affirmatives than by negatives; whereas it should duly and equally yield to both." And farther on: "The light of the understanding is not a dry or pure light, but drenched in the will and affections, and the intellect forms the sciences accordingly; for, what men desire should be true, they are most inclined to believe."

An attentive perusal of Dr. Gaulter's work will convince the reader that the doctor has not so far confined himself to the affirmatives as to reject the evidences having a contrary tendency: his is not a one-sided view of the question of contagion; and, should that question still continue to divide physicians into two opposing parties, it will not be the fault of Dr. G. that the parties do not join issue, but rather the fault of the word, or of the persons using it in a variety of meanings. It is this fallacy which has been the cause of violent controversies, not confined indeed to the medical profession. A word! what can appear more trifling, and yet, in its application, what can there be of greater importance? "Words," says the great writer whom we have just quoted, "are generally imposed according to vulgar conceptions, and divide things by lines that are most apparent to the understandings of the multitude; and, when a more acute understanding, or a more useful understanding, would remove these lines, to place them according to nature, words cry out, and forbid it. And hence it happens that great and serious disputes of learned men frequently terminate in controversies about words and terms, which it were better to begin with, according to the prudent method of the mathematicians, and reduce them to order by definitions."

Two months before the outbreak of the disease, an inspection of the town of Manchester was conducted under the orders of a well-organized board of health, and "disclosed in the quarters of the poor such scenes of filth, and crowding, and dilapidation—such habits of intemperance and low sensuality—and, in some districts, such unmitigated want and wretchedness, that the picture of the moral and physical state of the poor, which an active member of the board subsequently drew, deriving some of his darkest colours from the inspection, seemed to the minds of many among the more easy of the inhabitants as little less than a malicious libel on the town." (P. 2.) Fortunately, however, the disease was comparatively mild, although it was presumed, from the above statement, that the whole working population would have been swept away by the disease.

The first subject which our author considers is the "Origin of the Epidemic." The prevalence of cholera in the spring of 1832, and the extraordinary intercourse between Manchester and many great towns in the north of England and Scotland, where the disease was then prevailing, were regarded as sufficient reasons for the hourly expected outbreak of the disease at Manchester. The means taken to prevent its arrival are ludicrous enough.

"Two inspectors were set to watch the canal boats; a feeble array against an enemy that had the credit of having overmatched the armed cordons of Austria and Russia. At the same time the tramper was allowed to walk into the town unmolested; and the coaches, of which at least a hundred arrive daily, and the railroad, which at that time was pouring in weekly a tide of several thousand passengers, were left at perfect liberty to import what and whom they pleased." (P. 6.)

Such were the measures taken to prevent the introduction of the disease, and such the various means by which it might be introduced; and it is not to be regretted that more shackles were not placed on the commerce of the country. Our author compares the means taken to exclude the disease to the plan adopted by the country gentleman described by Addison, who thought to keep out the crows by nailing up his park-gate.

Dr. Gaulter is of opinion that all the precautionary measures which might have been adopted would have been followed by the same result: that the disease came not to Manchester by coach; that it neither sailed nor glided suddenly in; that it was not propelled by the force of steam. On the contrary, he says "it arose upon the spot." We will give the first case in his own words:

"On Thursday, the 17th of May, James Palfreyman, aged twenty-nine, a coach-painter, who lived in Somerset street, Dolefield, began to complain of nausea and pain of the bowels. He was seized at midnight, on Friday, with the characteristic symptoms of cholera, of which a clear and minute description, made from notes taken at each visit, was forwarded to the board of health by Mr. Stephens, the surgeon, for whose advice he applied. He died on Saturday afternoon, the 19th of May, at half-past two, in Coronation street, Salford, having been unthinkingly removed there by his family, who were changing their residence. No symptom during life, and no appearance after death, was wanting to mark this for a genuine case of malignant cholera. Palfreyman was a fine, stout, well-proportioned man; his house was not crowded; he was earning comfortable wages; the street where he lived was, in comparison with others, moderately clean and open. On the other hand, there was nearly opposite to his house a large dunghill attached to some extensive stables, (Wright's,) and Palfreyman had often complained of the fœtor which issued from the base of the wall behind which it was placed. Though his health was in general good, he had had repeated attacks of painter's colic; was subject to severe diarrhœa on taking weak acids; was an occasional drunkard; had been drunk the Tuesday night preceding the attack, and had eaten on Wednesday at dinner heartily of lamb's head, and what are called here the appurtenances, the liver, heart, &c. He had never been well after this meal. It was established, by the most diligent inquiry, that Palfreyman had had no communication, direct or indirect, with any infected person or thing." (P. 6.)

In this case the causes predisposing to cholera are sufficiently evident: whether the fœtor arising from the dunghill was sufficient of itself to produce the disease we will not venture to decide.

In Oldham the disease had likewise a doubtful origin; and in Bury, where only two cases occurred, the first was in a tramper, who did not communicate the disease, and the second in a respectable inhabitant, who had neither been exposed to contagion from the tramper, nor to any other source. Dr. Black, of Bolton, says that no case of the disease could be traced to importation into that town, nor had the first patients the least intercourse with any infected person or places. Dr.

Gaulter remarks, that, while "it is undoubtedly communicable from man to man, its dissemination over so large a portion of the habitable globe has been, on the whole, effected without contagion, by the successive springing up, sometimes in distant, but more commonly in adjacent places, of the poison, whatever this may be, that causes the disease. The more rigorously the evidence is examined, the more will the mind be confirmed in this conclusion, until it finally shakes off the idea of the contagious itineration of the disease; an idea founded, it must be admitted, on the most specious appearances, and strengthened by being associated with every term currently employed to express its progress." (P. 8.)

To the fallacy respecting the origin of the epidemic which our author has just mentioned, he adds a second, which has been the cause of much error, viz. the confident assumption of the importation of the disease, from the mere circumstance of its breaking out being coincident with the arrival of persons from an infected district; to this he adds a third source of fallacy, which he asserts has been more prolific of misrepresentation than either of the two already noticed: that is, the inference of contagious importation, from the occurrence of first cases either among the shipping of a seaport, or the parts of a town with which the shipping is in contact, or generally in places situated on the banks of a navigable river, although that inference has been drawn either without the prosecution of any inquiry into the history of those first cases, or in direct contradiction to the results of this inquiry. Dr. Gaulter has bestowed much labour in investigating the history of the disease as it has shown itself in various parts of the world. We must refer our readers to his work, if they desire to be acquainted with the arguments which he adduces in refutation of the opinions which we believe have been adopted by the majority with respect to the spreading of the epidemic. We will give the summary of his arguments in his own words:

"That the conclusion however is one of the utmost practical moment, cannot be denied. It is on the mode in which the disease originates, and not on that in which after its commencement it is subsequently propagated, that the important question of quarantine depends. The disease, after it has established itself in a given place, may be more or less contagious; but if the fact turns out to be that it arises in ninety-nine cases out of a hundred without importation—that imported cases of cholera, like imported cases of ague, prove barren—and that the disease spreads only after the appearance of spontaneous cases, quarantine is useless, and the injury it inflicts on the commercial relations and maritime intercourse of the country that adopts it is an ábsolute and uncompensated evil. So satisfied I am of the truth of this conclusion from an extended examination of the history of first cases, that I durst almost venture to assert of cholera what Dr. Vetch boldly but truly asserted of yellow fever, (which in its causes differs chiefly from cholera in this, that a high atmospheric temperature is indispensably necessary for the existence of the former, while it only favours that of the latter,) that if the government were to offer a premium for the introduction of cholera into any town selected for that purpose, it could not be introduced. Springing up upon the spot, on the one hand no amount of intercourse can certainly convey the poison so as to give rise to a genuine eruption of the disease, and on the other hand no human artifices of exclusion can, in the very nature of things, shut it out." (P. 33.)

The second division of the work is headed "Extension of the Disease by Spontaneous Cases." Our author says, that

"If the first cases in Manchester were spontaneous, the presumption undoubtedly is that other cases, in the progress of the epidemic, would bear the same character; and accordingly, by a reference to the tables, it will be seen that, of the 200 edited cases, there are 117 where, after the most searching and jealous inquiry, no communication could be traced; and, of the 100 unedited, there are 62 more." (P. 34.)

We think we shall have discharged our duty to our readers by referring them to the chain of evidence which Dr. Gaulter has adduced: to bring forward any individual case would be a means of throwing but little light on the question.

The like observations will apply to what our author has essayed under the head of "Extension of the Disease by Contagious Cases." We have only to remark, that the question of contagion and non-contagion is handled by Dr. G. in a very able manner: he has not given up his mind to affirmatives without duly weighing the negatives; and, should his train of reasoning fail to carry conviction to the reader, his arguments will at least afford much pleasure, from the skill and ability with which they are conducted.

"On the Causes of the Disease, and chiefly its Secondary Causes," our author remarks, "There is therefore, at all events, nothing contradictory to the hypothesis, which, the more I reflect upon it, appears to me to be the more probable, that the miasm which produces the spontaneous attacks of cholera is precisely the same matter as the poison which, in contagion, transmits the disease." (P. 94.) This hypothesis he supports by a series of arguments, which are too long to admit of being extracted.

A large share of the exemption from cholera which the working classes at Manchester enjoyed during the epidemic, Dr. Gaulter is disposed to ascribe to the cotton-factories in which they work. "Out of the 200 tabular cases, only 23 worked in factories, and of these, 12 were out of work, or accidentally remaining at home." (P. 120.) A like exemption of the factory people in actual employment was observed at Warrington, Stockport, and other manufacturing towns. How far the employment of the mind may have tended to produce this exemption from the disease we have not sufficient data to assert; at all events, we have the authority of Dr. Gaulter for stating that the men thought themselves safe from the disease in the factories, and spent no more of their time at home than was absolutely necessary.

Passing over the "Generating, Predisposing, and Exciting Causes," respecting which we may suppose our readers fully acquainted, and just hinting that they will find some useful information "on the best General Arrangements for meeting the Disease," we arrive at the "Treatment of the Disease." Our author remarks, "It happens not unfrequently in medicine that the pathology and treatment of a disease do not keep pace with each other. Of this, cholera is unhappily an example." After making some sensible remarks on the pathology of the disease, he says, "The saline remedies of Dr. Stevens were not confided in, having been found inefficient in the stage of collapse, and inferior to the ordinary practice in the incipient stage." (P. 148.) The cold-water plan of Dr. Shute was adopted in two or three cases, but without success; and venous injection was tried in eight cases, all of which terminated fatally. The tartar-emetic treatment, as described in a letter from Mr. Langford to Dr. Gaulter, appears to have been the one most in use at Manchester.

"The antimonial plan is as follows: Dissolve ten grains of tartarized antimony in seven and a half ounces of distilled water, with half an ounce of rectified spirit, of which give half an ounce every two hours, until the biliary and urinary secretions are properly restored; then gradually diminish its frequency. Allow toast and water, or whey, *ad libitum;* prohibit all heat and frictions, and give no other remedy. In all the pulseless cases, and when the pulse became feeble, I applied externally cloths dipped in warm spirit of turpentine, in preference to mustard poultices, as often as six times in twenty-four hours, over the thorax and abdomen, producing considerable cuticular excitement without vesication." (P. 152.)

The success which attended this plan of treatment was not so great as that which was obtained by the administration of calomel, as recommended by Dr. Ayre; in 80 cases there were 53 recoveries, and 27 deaths. The consecutive symptoms were treated much after the usual manner. We subjoin a list of the cases as they occurred in Manchester and the adjoining townships.

*Cholera in the Township of Manchester. Population,* 142,026.
Attacked, 1325. Died, 674. Recovered, 624. Unreported, 27.

*Cholera in Salford. Population,* 40,786.
— 700. — 216. — 474.

*Cholera in Chorlton upon Medlock. Population,* 20,569.
— 88. — 34. — 52.

*Cholera in Stockport. Population,* 25,469.
— 72. — 32. — 38. Unreported, 2.

The epidemic cholera, which arose in Manchester a little earlier than the common cholera, sprang to its climax by a sudden and very remarkable leap in the month of August, in which, according to Dr. Dalton's journal, more rain fell than in any month during the whole year. An extract from the parish register of Stockport "shews the singular, and I believe unique fact, that the mortality of the months included was considerably greater in that town during the year preceding that of the cholera, than during the cholera year itself." (P. 159.) If our author had consulted the registers of the different parishes in and about London, we think he would have found the fact above mentioned not so "singular and unique" as he imagines.

This laborious work contains an account of the first two hundred cases of cholera which appeared at Manchester, a part which will be consulted with advantage; but the value of the book would have been doubled had our author informed us where he obtained the materials for it. There is no preface; and we are left to guess how a physician, practising at Chorlton-upon-Medlock, could be acquainted with the minutest details of cases occurring at Manchester.

